# Using metagenomic analysis to assess the effectiveness of oral health promotion interventions in reducing risk for pneumonia among patients with stroke in acute phase: study protocol for a randomized controlled trial

**DOI:** 10.1186/s13063-020-04528-3

**Published:** 2020-07-10

**Authors:** Juncang Wu, Yuanchang Dai, Edward C. M. Lo, Yinliang Qi, Ya Zhang, Quan-li Li, Ruoxi Dai

**Affiliations:** 1 The Second People’s Hospital of Hefei, Hefei Hospital Affiliated to Anhui Medical University, 246 Heping Road, Hefei, China; 2 Department of Dental Public Health, Faculty of Dentistry, The University of Hong Kong, 34 Hospital Road, Sai Ying Pun, Hong Kong; 3 Key Laboratory of Oral Diseases Research of Anhui Province, Stomatological Hospital & College, Anhui Medical University, Hefei, China

**Keywords:** Metagenomic, Next-generation sequencing, Pneumonia complicating stroke, Oral health promotion, Randomized controlled trial

## Abstract

**Background:**

The prevalence of pneumonia complicating stroke in acute phase has a poor prognosis and higher risk for death. Oral opportunistic pathogens have been reported to be associated with pneumonia among people with compromised health. Oral health promotion is effective in reducing dental plaque among patients with stroke, which is considered as reservoirs for oral opportunistic pathogens. This study evaluates the effectiveness of oral health promotions in reducing the prevalence of pneumonia via its effects on composition and relative abundance of oral opportunistic pathogens.

**Methods/design:**

This study is a randomized, single-blind, parallel trial of 6 months duration. The study is being conducted at one of the largest medical teaching hospitals in Hefei, China. A total of 166 patients with stroke and free from any post-stroke complication will be recruited. After enrollment, patients will be randomized to one of the following groups: (1) oral hygiene instruction (OHI) or (2) OHI, 6-month use of powered tooth brushing, and 0.2% chlorhexidine gluconate mouth rinse (10 ml twice daily). The primary outcome is the prevalence of pneumonia complicating stroke. Patients will be monitored closely for any occurrence of pneumonia over the entire period of this trial. Oral rinse samples will be collected at baseline and multiple follow-up reviews (3, 5, 7 days, and 1, 3, 6 months after baseline). Next-generation sequencing will be employed to detect composition and relative abundances of the microorganism in the oral rinse samples. Questionnaire interviews and clinical oral examinations will be conducted at baseline and 1, 3, and 6 months after baseline.

**Discussion:**

The findings of this trial will provide evidence whether oral health promotion intervention is effective in reducing the prevalence of pneumonia complicating stroke via its effect on the oral microbiome. The analysis of the outcomes of this trial is empowered by metagenomic analysis at 16S rRNA level, which is more sensitive and comprehensive to help us detect how oral health promotion inventions affect the oral microbiome in terms of its composition, relative abundance, and interactions between species, which all may contribute to the occurrence of pneumonia complicating stroke.

**Trial registration:**

ClinicalTrials.gov NCT04095780. Registered on 19 September 2019

## Background

Stroke is defined as a “rapidly developing clinical signs of focal (or global) disturbance of cerebral function, with symptoms lasting 24 h or longer, or leading to death, with no apparent cause other than of vascular origin” [1]. A systematic review published in *The Lancet* (2010) reported that no significant change in the incidence and mortality-to-incidence ratios (MIR) of ischemic stroke has occurred during the last two decades [2], while in contrast, a significant increase of hemorrhagic stroke by 22% (95% CI 5 to 30%) and reduction in MIR by 36% (95% CI 16–49%) were observed in low- and middle-income countries [2]. China has the highest incidence rate of hemorrhagic stroke in the world with 159.81 per 100,000 person-years [2]. As a result of the increased incidence and decreased MIR, greater disease burden would be placed on countries with limited financial resources due to a greater number of stroke survivors and disability-adjusted life years (DALYs) [3].

Following stroke, 85% of the patients have limb dysfunction in acute phase, which makes them unable to perform oral self-care [4]. A meta-analysis found that compared to healthy controls, patients with stroke had significantly increased DMFT, plaque index, probing depth, and clinical attachment loss [5]. Another systematic review summarized that following stroke, patients have reduced lip force and tongue pressure against hard palate and uncoordinated masticatory muscle movement, all of which collectively lead to the difficulties of identifying the location of food bolus and propelling it into the esophagus [6]. Food debris is more likely to be accumulated in the oral cavity, giving rise to the growth of opportunistic pathogens (aerobic and facultatively anaerobic Gram-negative bacilli, yeasts, *Staphylococcus aureus*). Among those hospitalized patients with stroke in Hong Kong, aerobic and facultatively anaerobic Gram-negative bacilli (AGNB) was isolated among 72.8% of them [7], and yeast was isolated among 55.4 to 59.3% of them [7, 8]. Among those patients with stroke during the rehabilitation period, the prevalence of AGNB still maintains at the level of 47.9% and yeasts at the level of 50.5% [9]. All the aforementioned reflected a higher prevalence of opportunistic pathogens isolated from patients with stroke than healthy subjects [10]. It has been reported that those opportunistic pathogens are associated with aspiration pneumonia. One study recovered pathogens from the protective bronchoalveolar lavage fluid (PBAL) and compared those pathogens to those isolated from dental plaque by pulsed-field gel electrophoresis (PFGE). A number of *S. aureus* and Gram-negative bacilli isolates in PBAL matched genetically those from the corresponding dental plaque [11]. It is thus posited that the oral cavity can be a reservoir of respiratory pathogens responsible for pneumonia among those people with systemic diseases.

The prevalence of pneumonia complicating stroke in acute phase is ~ 20 to 60% [12, 13]. Although it is not the most frequent medical complications following stroke, it may be the most difficult to manage and has a very poor prognosis, leading to a threefold risk of death [14, 15]. Although it occurs most often within 1 week after the onset of stroke, yet it can still develop in 6 to 30 months after stroke [13]. Prophylactic use of antibiotics to reduce the incidence rate of stroke-associated pneumonia has proved to be of little use in a multi-center randomized controlled trial [16]. Therefore, new preventive approaches for stroke-associated pneumonia aiming at reducing respiratory pathogens in the oral cavity are urgently needed.

Oral health promotion is a range of activities which enable individuals and communities to increase control over the determinants of oral health and thereby improve their oral health. Since dental plaque is associated with many oral diseases, the major composition of oral health promotion interventions aims at removing dental plaque. Dental plaque removal measures include mechanical and chemical measures. Powered tooth brushing has been proved to be more effective than manual tooth brushing in reducing plaque and gingivitis [17]. Chlorhexidine is the gold standard among those chemical plaque control measures, and its antiplaque effectiveness has been well established [18]; however, its effectiveness against oral opportunistic pathogens in the clinical setting is unclear [9].

Moreover, oral health-related quality of life (OHRQoL) reflecting an interaction between oral health conditions and their contextual cultural factors is now recognized as an important parameter in outcome assessment of oral health in epidemiological studies and clinical trials. OHRQoL consists of functional (biting, chewing, swallowing, and speaking) and psychosocial wellbeing (concerns about certain oral conditions and satisfaction with respect to oral care and self-image of appearance) [19]. Therefore, it is also critical to assess the effectiveness of oral health promotion intervention in improving subjective oral health among patients with stroke.

Next-generation sequencing (NGS) technique at the 16S rRNA level will be employed in this proposed trial to assess the change of the composition and relative abundance in the oral microbial community. NGS technique enables us to sequence a great number of genes at one time and profile taxonomic composition at the genus or species level with a deeper coverage of microbial community [20].

We proposed this randomized controlled, parallel, single-blinded, equivalence trial with an allocation ratio of 1:1, which compares the effectiveness of powered tooth brushing with adjunctive use of chlorhexidine mouth rinsing and oral health instruction (OHI) versus OHI in (1) reducing the prevalence of aspiration pneumonia complicating stroke, (2) changing the composition and relative abundance in the oral microbial community, (3) improving oral hygiene status in terms of reducing dental plaque and gingival bleeding, and (4) improving oral health-/generic health-related quality of life.

## Method

### Participants

This study is a randomized, single-blind, parallel-group controlled trial of 6 months duration. Follow-up reviews will be conducted in 3, 5, and 7 days, and 1, 3, and 6 months after enrollment. Multiple calls will be made 1 week before each follow-up visit to remind participants of their visit. The study was approved by the Biomedical Ethics Committee of Anhui Medical University (reference number: 20170095, see additional file 1) and registered at the ClinicalTrials.gov provided by the US National Library of Medicine (Registration No.: NCT04095780). The protocol was written according to the SPIRIT Checklist (Standard Protocol Items: Recommendations for Interventional Trials, see additional file 2).

The study is being conducted at The Second People’s Hospital of Hefei, Hefei Hospital Affiliated to Anhui Medical University, Hefei, China. This hospital is one of the largest general hospitals in Hefei, which is the capital city of Anhui province. Patients with stroke in acute phase receive care at the Inpatient Stroke Unit, Department of Neurology, and will form the study subjects for this trial. During their inpatient stay in the Stroke Unit, they receive multidisciplinary treatments and support involving physiotherapy, occupational and speech therapy, and social work service. The length of hospitalization is usually 1 week. After discharge, patients with stroke are scheduled for regular follow-ups and outpatient rehabilitation. Our oral health research team is collaborating with the Stroke Unit in helping with oral care for those patients following stroke.

The following selection criteria are applied: (1) having onset of stroke within 3 days and free of any post-stroke complication; (2) having moderate to severe functional disability—Barthel Index (BI) scores of < 70; (3) being conscious and respiring voluntarily without ventilator; (4) not having any lung disease and lower respiratory infection; (5) not having indwelling naso-gastric feeding tubes; (6) having dysphagia as shown by Gugging Swallowing Screen (GUSS) test; (7) having normal cognitive ability or mild impairment—Mini-Mental State Examination (MMSE) > 18; (7) having the ability to follow instructions (as an assessment of compliance of oral health intervention); (8) not having systemic administration of antibiotics; and (9) not being edentulous. Patients who meet the above selection criteria will be provided with detailed information regarding potential benefits and adverse effects of the interventions, the procedures of this trial, and the anticipated outcomes. They will be asked by a researcher in our team to sign on a written informed consent before enrolling in this study (written informed consent, additional file 3).

According previous studies [12, 13], pneumonia complicating stroke usually occurs within 1 week after stroke. We set the prevalence of pneumonia within 1 week after onset of stroke as the primary outcome of this study since we would like to explore whether oral health promotion interventions could possibly reduce the prevalence of pneumonia after stroke. According to previous epidemiological studies, the prevalence of pneumonia complicating stroke is 20 to 60% [12, 13]. Thus, we hypothesize that in the comparison group, the prevalence of pneumonia complicating stroke within 1 week is 40%, while the prevalence could be decreased to the lower limit of 20% in the interventional group. We set the statistical power at 80% and the statistical significance level at 0.05. Anticipating a 10% dropout rate over the first week of this clinical trial, the initial sample size for each group is proposed as 83 patients per group (166 subjects in total, Gpower 3.0.10 statistical software).

### Interventions

Participants will be block randomized to one to the two groups with a block size of 4: (1) comparison group—oral hygiene instruction (OHI)—or (2) interventional group—powered tooth brushing, 0.2% chlorhexidine gluconate mouth rinse (10 ml twice daily), and OHI. This clinical trial is single blinded with researchers blinded to the allocations while subjects are aware of it. A researcher in our oral health research team who is not involved in recruiting participants or assessing outcomes will use SPSS 25 for Windows (SPSS Inc., Chicago, USA) to generate the randomized sequence. Another researcher in our team who is responsible for participant screening/recruitment and outcome assessment will be blinded to the treatment assignment until the end of this trial. The allocation sequence number will be concealed in an opaque envelope and provided to a staff at the stroke unit who is independent of our team. The staff is responsible for assigning subjects to one of the two groups after enrollment. After the end of this study, the staff will reveal the allocation sequence. Participants in the interventional group will be asked to bring back their powered toothbrush and empty bottles of chlorhexidine mouthwash for replacement at follow-up visits after discharge. This strategy may improve adherence to intervention protocols.

Immediately after group assignment, the subjects and their primary caregivers in the interventional group will be invited to a one-to-one workshop provided by a dental professional regarding oral hygiene. This content of this workshop includes the importance of oral health following stroke and how to implement standardized oral hygiene care (Bass brushing technique, flossing, mouthwash use, and cleaning partial dentures). The primary caregivers will be responsible for carrying out the oral hygiene care for the patients with stroke. After the demonstration, the primary caregivers will be asked to perform their oral hygiene care skills and will be corrected by the dental professional if oral health care is not conducted appropriately. For those in the interventional group, they will be provided with a powered toothbrush and its manufacturer instructions. In addition, they will be given 0.2% chlorhexidine gluconate mouthwash. They will be asked to use a powered toothbrush to brush their teeth and rinse twice daily with 10 ml of the mouth rinse (at least 30 min after brushing). For those subjects in the comparison group, they will be only provided with oral hygiene instruction in the one-to-one workshop. Due to ethical considerations, those subjects in the comparison group will be given the same oral hygiene products after the completion of the clinical trial. Participants may withdraw from this study at any time points. Participants who will have systemic antibiotic use longer than 1 week after interventions will be excluded from this study.

### Outcomes

**Fig. 1:**
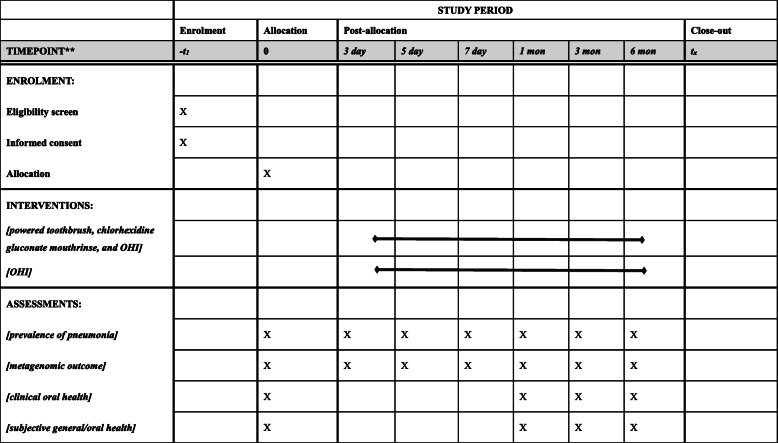
The schedule of enrolment, interventions, and assessments

The primary outcome is the prevalence of pneumonia complicating stroke within 1 week after baseline. The secondary outcomes include the following: (1) the prevalence of pneumonia complicating stroke within 1, 3, and 6 months after baseline; (2) metagenomic outcomes in terms of composition and relative abundance of oral microbiome in oral rinse samples in 3, 5, and 7 days, and 1, 3, and 6 months after baseline; (3) oral hygiene status and gingival bleeding in 1, 3, and 6 months after baseline; and (4) generic health-/oral health-related quality of life in 1, 3, and 6 months after baseline. The time point for assessing each outcome is illustrated in Fig. 1.

#### Assessment of pneumonia complicating stroke

We will employ the diagnostic criteria of pneumonia complicating stroke developed by the Stroke Consensus Group [21]. The recommended diagnostic criteria for definite and probable stroke-associated pneumonia in patients not receiving mechanical ventilation are as follows. Patient who is diagnosed as definitive case should meet at least 1 of the following: (1) fever (> 38 °C) with no other recognized cause, (2) leukopenia (< 4000 WBC/mm^3^) or leukocytosis (> 12,000 WBC/mm^3^), and (3) for adults ≥ 70 years old, altered mental status with no other recognized cause. And patient should also meet at least 2 of the following: (1) new onset of purulent sputum, change in character of sputum over a 24-h period, increased respiratory secretions, or increased suctioning requirements; (2) new onset or worsening cough, dyspnea, or tachypnea (respiratory rate > 25/min); (3) rales, crackles, or bronchial breath sounds, and (4) worsening gas exchange. Patient should also have no less than 2 serial chest radiographs (CXR) with at least 1 showing new or progressive and persistent infiltrate, consolidation, or cavitation. Probable case also needs to meet all the criteria except that diagnostic changes on the initial CXR and repeat CXR (or where CXR not undertaken) are absent, and no alternative explanation or diagnosis exists. Since most patients are hospitalized within 1 week in this study, it would be feasible for us to diagnose any pneumonia occurring in this period, but it would be challenging to diagnose whether they develop any pneumonia after hospital discharge. We will inquire their medical history and use their medical notes to make diagnosis at their follow-up visits after discharge.

#### Metagenomic outcome assessment

Patients will be instructed to rinse his/her mouth for 60 s with 10 ml of sterile phosphate-buffered saline (PBS, 0.1 M, pH 7.2) and to spit the rinse into a sterile container. The rinsed samples will be centrifuged for 10 min (1700*g*, 4 °C). The pellet will be re-suspended in 1 ml sterile PBS, from which total genomic DNA will then be extracted. Optimized PCR primers will be utilized for amplifying the targeted region (such as V3–V4) of the bacterial 16S rRNA gene. The 16S rRNA amplicons will be isolated and purified for multiplexing high-throughput DNA sequencing. High-quality reads will be classified into different taxonomic groups based on the Human Oral Microbiome Database with a default confidence threshold. To control the sequencing effort across samples within a study, all microbiome data will be rarefied at an acceptable sequencing depth. The within-sample diversity of microbiomes will be assessed by alpha diversity measures such as the Shannon index. Beta diversity measures the overall microbiome similarity between each pair of samples, generating a distance matrix between all pairs of samples within the study. Principal coordinate analysis (PCoA) based on a distance matrix (such as UniFrac or Bray-Curtis) is commonly used to visualize beta diversity data in an unsupervised manner. To determine differences in the relative abundances, classical statistical methods/tools (e.g., Student’s *t* test, ANOVA) or linear discriminant analysis (LDA) effect size (LEfSe) will be used [22].

#### Clinical oral health assessment

Oral hygiene status will be assessed by Silness and Löe Plaque Index (PI) [23]. The PI will be assessed at six sites per tooth and charted on all permanent teeth. The criteria for the Silness and Löe PI are as follows: 0 = no plaque detected with probe, 1 = plaque not visible by unaided eye but detectable with probe, 2 = moderate amount of plaque, and 3 = abundance of plaque. The percentage of tooth sites with moderate to abundant plaque (PI score, 2 or 3) will be calculated for each subject. Gingival bleeding will be assessed by the Gingival Bleeding Index (GBI) [24]. The GBI will be charted on all permanent teeth and assessed at six sites per tooth. The criteria for GBI are 0 = no bleeding after probing and 1 = presence of bleeding within 10 s after probing. The percentage of bleeding sites (GBI score, 1) will be calculated for each subject. At each study visit, ~ 10% of the subjects will be randomly selected for the reevaluation of the PI and GBI and the intra-rater reliabilities will be evaluated. Other clinical oral health outcomes including dental caries experience (DMFT), oral mucosa conditions, and dental prosthesis status will be recorded according to WHO Basic Oral Health Survey Guidelines [25].

#### Subjective health assessments

The subjective health assessment includes generic health-related quality of life (HRQoL) and oral health-related quality of life (OHRQoL) assessments. Patients will be interviewed with Chinese versions of the Short Form Health Survey 12 (SF-12) [26], the Oral Health Impact Profile 14 (OHIP-14) [27], and the Geriatric Oral Health Assessment Index (GOHAI) [28]. The SF-12 consists of 12 items, and each of them has its own physical component summary (PCS) and mental component summary (MCS) regression coefficients [29]. The response to each item will be multiplied by its PCS regression coefficient and added together with the PCS constant to provide Physical Health summary scores (SF-12 PCS). Mental Health summary scores (SF-12 MCS) will be calculated likewise. For OHIP, responses to the frequency of an event occurring as described by each item are coded on a 5-point Likert scale: 0 = never, 1 = hardly ever, 2 = occasionally, 3 = fairly often, and 4 = very often/all of the time. Summary OHIP-14 score and domain scores will be derived by summating responses to each item (i.e., scores 2, 3, and 4) [30]. For GOHAI, responses to the frequency of an event occurring as described by each item are coded using a 5-point Likert scale: 1 = always, 2 = often, 3 = sometimes, 4 = seldom, and 5 = never. Summary GOHAI scores will be derived by summating responses to items after reversing the coding of the three positively worded items (swallowing, appearance, and discomfort when eating) [31].

#### Collection of other confounding factors

Information will be also recorded regarding sociodemographic profiles (age, gender, education level, working status, and social pension), history of smoking, tooth brushing habit, time since last dental visit, dominant hand function as assessed by grip strength, cognitive ability as assessed by Mini-Mental State Examination [32], and number of comorbidities.

#### Data management

The principal investigator will oversee the data collection, entry, analyses, and reporting. Two researchers in our team will conduct data proofreading after the completion of data entry. The data will be securely stored in SPSS files in a computer with a password. The statistics software packages SPSS 25 for Windows (SPSS Inc., Chicago, USA) and QIIME 2 will be used to analyze the data. Per-protocol (PP) will be employed for both primary and secondary outcomes in bivariate analyses. Within- and between-group comparisons will be conducted over the clinical trial period. When the outcome variables are continuous and follow a normal distribution, a paired *t* test for related samples will be performed to determine the significant differences over time within a group. The Student *t* test for independent samples will be performed to compare the mean of the outcomes between the groups at baseline and follow-up reviews. When the outcome variables are continuous but do not follow a normal distribution, the Wilcoxon signed-rank test will be performed to determine the significant differences over time within a group. The Mann-Whitney *U* test will be employed to compare the differences in the rank of outcomes between the groups at baseline and follow-up reviews. When the outcome variables are categorical, the McNemar test will be performed to identify the changes in prevalence over time within a group. The chi-square test will be employed to compare the variations in prevalence between the groups at baseline and follow-up reviews. Regression analyses will be employed to address the effect of confounding factors for outcomes. When conducting the regression analysis, the method of Last Observation Carried Forward (LOCF) will be employed to deal with missing outcomes at follow-up reviews. Linear regression will be adopted when the dependent variables are continuous, and the residuals of the regression followed a normal distribution. Negative binomial regression will be adopted when the distribution of the continuous dependent variables is over-dispersed. Logistic regression will be adopted when the dependent variables are binary.

## Discussion

Up until now, only six trials regarding oral health promotions among patients with stroke have been conducted; none of which was conducted in mainland China [33–38]. The oral health promotion interventions among patients with stroke in those studies were quite heterogeneous. Also, the outcomes in those studies mainly focused on the clinical aspect of oral health rather than general wellbeing. Among them, only two studies collected oral microbial samples to test the oral carriage rate and viable counts of oral opportunistic pathogens and assessed the prevalence of pneumonia as one of the outcomes [7, 9]; however, neither of them was able to associate the occurrence of pneumonia with the prevalence and load of oral opportunistic pathogens due to small sample size. Similar studies have also been conducted to evaluate the effectiveness of oral health promotions in reducing the risk for ventilator-associated pneumonia (VAP) among critically ill patients. One Cochrane systematic review published in 2016 summarized all the RCTs of oral health promotion interventions on VAP and found that oral health promotion interventions including chlorhexidine mouthwash or gel reduce the risk of developing VAP in critically ill patients from 25 to about 19% [39]. Again, these findings were only based on clinical outcomes which is the diagnosis of VAP. Although the clinical diagnosis is the gold standard to confirm cases of pneumonia, it is not sensitive to provide indications for close monitoring or early interventions yet.

It has been proved that oral opportunistic pathogens and aspiration pneumonia have a strong association [11], yet the ways to detect oral opportunistic pathogens in the aforementioned studies were limited in conventional culture and PFGE. The traditional culture method uses several selective culture media and is only able to detect those species which can be cultured. Presumptively, the identification of pure isolates of species on the selective culture medium is based on colony morphology and the Gram stain method, which largely depends on personal experience and subjective judgment. PFGE can only target several suspicious species and identify them qualitatively. Unlike those conventional techniques, NGS is able to assess the phylogenic structure, relative abundance, and correlation network of the whole oral microbial community. The high-throughput sequencing of 16S rRNA genes can usually provide taxonomic resolution of the whole microbial community at the genus or even species level. In this trial, we will employ the change of composition, relative abundance, and correlation network from metagenomic analysis as part of the outcome measures. Compared to conventional microbiological techniques, the outcomes from the metagenomic analysis are more comprehensive and sensitive to reflect how the whole oral microbial community is affected by the oral health promotion interventions, and how the change of the whole oral microbial community is associated with the risk for pneumonia complicating stroke. In this trial, the oral rinse samples will be collected at baseline and multiple times within 1 week after enrollment. These consecutive samples in a short time interval will provide a close observation of the dynamic change of oral microbial community, and possibly reflect the exact status of the oral microbial community before the event of pneumonia if any participant develops pneumonia. The findings of this trial will provide much-needed evidence for medical professionals in the field of cardiovascular diseases to advocate or disregard the inclusion of oral health promotion interventions into multidisciplinary stroke treatment.

## Supplementary information

**Additional file 1.** IRB approval.

**Additional file 2.** SPIRIT 2013 Checklist: Recommended items to address in a clinical trial protocol and related documents.

**Additional file 3.** Explanatory note for potential participant and consent form.

**Additional file 4.** Translator’s certification and Anhui Province Natural Science Foundation Proposal of Funding Project.

## Data Availability

Dr. Ruoxi Dai will have access to the final trial dataset and disclose the randomized sequence.
