# Supplementary material for: Using metagenomic analysis to assess the effectiveness of oral health promotion interventions in reducing risk for pneumonia among patients with stroke in acute phase: study protocol for a randomized controlled trial
Source: Trials. 2020 Jul 10;21:634. doi: 10.1186/s13063-020-04528-3 (PMC7350693; doi:10.1186/s13063-020-04528-3)
Supplement: Supplementary file 1 — Additional file 1. IRB approval. [file 13063_2020_4528_MOESM1_ESM.pdf]

NO. 20170095

Biomedical Ethics Committee of Anhui Medical University
Address: 81 Meishan Road, Hefei, China

Principal: Ruoxi Dai
Protocol title: The effectiveness of oral health promotion on pneumonia complicating stroke
Institute: Anhui Medical University

1. The ethics committee has approved the study protocol, and discussed the following three aspects in particular:
· The rights and interests of the participants.
· Measures to ensure to obtain informed consent.
· The potential risks and benefits.

2. The study protocol has been approved. In the implementation process, please use approved informed consent, questionnaires and explanatory note, etc.

3. Do not deviate from or make changes to the study protocol without prior written committee approval, except when it is necessary to eliminate immediate hazards to research subjects or when the change involves only logistical or administrative issues.

4. Any adverse reactions during the study shall be reported to the ethics committee in written forms immediately.

Chairman: Qixing Zhu

Signature:

安徽医科大学生物医学伦理委员会
（盖 章）

Effective date: 03/01/2017

编 号：20170095

安徽医科大学生物医学伦理委员会
课题论证报告
（正　本）

课题负责人：戴若曦

课题名称：口腔健康促进对脑卒中肺炎并发症作用的研究

承担单位：安徽医科大学

一、伦理委员会对该课题方案进行了论证，并特别对以下三方面进行了认真讨论：
1、研究对象的权利与利益；
2、确保取得知情同意的措施；
3、存在的危险与可能的受益。

二、同意实施该课题方案。实施过程中请使用经论证的知情同意书、问卷、说明信等材料。

三、课题方案如需修改，须事先经伦理委员会论证方可实施，修改内容及其原因需详细备案。

四、实施过程中如出现任何不良反应需立即向伦理委员会做出书面报告。

主席姓名：朱启星　　　主席签字：朱启星

生效日期：2017 年 3 月 1 日

安徽医科大学生物医学伦理委员会
（盖 章）
