# Supplementary material for: Using metagenomic analysis to assess the effectiveness of oral health promotion interventions in reducing risk for pneumonia among patients with stroke in acute phase: study protocol for a randomized controlled trial
Source: Trials. 2020 Jul 10;21:634. doi: 10.1186/s13063-020-04528-3 (PMC7350693; doi:10.1186/s13063-020-04528-3)
Supplement: Supplementary file 3 — Additional file 3. Explanatory note for potential participant and consent form. [file 13063_2020_4528_MOESM3_ESM.docx]

**EXPLANATOTR NOTE FOR POTENTIAL PARTICIPANTS**

Invitation to join a medical research project:

*The effectiveness of oral health promotion on pneumonia complicating stroke*

Research conducted by:

The Second People’s Hospital of Hefei, Hefei Hospital Affiliated to Anhui Medical University

Thank you for agreeing to listen to an explanation of a clinical research study that we are doing at The Second People’s Hospital of Hefei.

You have recently suffered a stroke and ongoing a course of treatment as an in-patient. Although you will be discharged in around one week, you will continue to receive regular treatment as an out-patient.

People who are recovering from stroke have different levels of physical condition and well-being. Often after stroke it may be difficult for you to clean your teeth and keep your mouth fresh. Sometimes it is also more difficult to chew and swallow food. We are currently investigating different forms of treatment to improve the oral health condition of people with stroke. We are particularly interested in your feeling about the treatments, how they affect your mouth and teeth and your daily living.

We would like to invite you to take part in our study. If you would like to help us it would involve the following treatment and assessments:

First of all, during your stroke treatment in the hospital, we would invite you to participate in a one-week clinical study. Then, we would like to see you again when you return to the hospital for a routine medical review appointment 1 month, 3 months and 6 months after you have been discharged from hospital.

If you would like to take part, first of all a dentist/research assistant would ask you a small number of questions about your general health, how you are feeling about yourself, and how the condition of your mouth and teeth feel. The questions are standard ones that have been used in similar studies in many parts of the world. They will allow us to assess your general well-being and the effect of any oral problems on your general health and quality of life. Thereafter you will be asked to rinse your mouth with some salty water and spit it in a small cup. Then, we would examine your mouth and check your teeth, examine your gums and inspect any dentures that you may wear. There would be no discomfort associated with the examination. You would be allocated randomly to one of two treatment groups. Depending on the treatment group, you would receive either: (1) professional advice on how to clean your teeth and dentures (if you have them) effectively in daily life, or (2) professional advice on how to clean your teeth and dentures (if you have them) effectively using a powered toothbrush and a Chlorhexidine mouthrinse twice daily for 6 month period. When you come back at 1, 3, and 6 months, we would do the same procedures again.

By doing this study we hope to improve the oral health condition of patients with stroke and to identify which type of treatment is the most effective in reducing potential risk for pneumonia complicating stroke. We would then be able to improve our methods of assisting patient with stroke in their general recovery.

Side effects: There are no known major side effects of using Chlorhexidine mouth rinse. However, the mouth rinse may temporarily stain the teeth and can be removed by prophy. Also some people find the taste bitter.

**You may stop the treatment at any time if you wish.**

Benefit: It is likely that you will have direct benefit in terms of improvement in your oral health condition by taking any of the two treatments, which potentially decrease the risk for developing pneumonia following stroke.

Confidentially: The information you provide will be treated in the strictest confidence. Results of this research study may be presented ay scientific meetings or in scientific publications. However, your identity will not be disclosed in any of the presentations or publications.

New findings: You will be told of any significant new findings developed during the course of this study which may affect your willingness to continue your participation.

**For further information please contact:**

Dr. Yinliang Qi, The Second People’s Hospital of Hefei, Hefei Hospital Affiliated to Anhui Medical University, Hefei, China. Tel. 189 5513 3681

We are very grateful for your help.

CONSENT FORM

for you to take part in the research project:

*The effectiveness of oral health promotion on pneumonia complicating stroke*

Research conducted by:

The Second People’s Hospital of Hefei, Hefei Hospital Affiliated to Anhui Medical University

The following statements are to check that you understand and consent to the procedures involved in taking part in this research:

1. I confirm that I have read and understood (or had someone read and explain) the information leaflet for the above study and have been given a copy to keep. I have had the opportunity to ask questions about the project and I understand why the research is being done and any risks involved.
2. I understand that my participation is voluntary.
3. I agree to take part in the study.
4. I understand that all information that I provide, or that is passed on from my doctor to the research team will be kept confidential and only the research team will see it.
5. I understand how the data will be collected, that giving data for this research is voluntary and that I am free to withdraw my approval for use of the sample at any time, without giving reason and without my medical treatment or legal rights being affected.
6. I understand that I will not benefit financially if this research leads to the development of a new treatment or medical test.
7. I understand that I am free to withdraw from the study at any time, without giving reason and without my medical treatment or legal rights being affected in any way.

**Please sign and date this Consent Form below:**

Name of Subject or Proxy* in BLOCK letters Date Signature

Name of Investigator in BLOCK letters Date Signature

Name of Witness in BLOCK letters Date Signature

**If patient agrees by assent but is unable to write, then a proxy will sign on his/her behalf.*

**对可能参加研究的说明**

研究专案：**口腔健康促进对脑卒中肺炎并发症作用的研究**

研究实施者：合肥市第二人民医院，安徽医科大学附属合肥医院

感谢您同意听取我们将在安徽医科大学和合肥市第二人民医院进行的一项临床研究的说明。

您最近遭遇了一次中风，并已住院接受治疗。虽然您将在大约一周后出院，但在出院以后将继续接收常规治疗。

中风患者身体情况和健康状况可能会有差异。通常中风后患者在自己清洁口腔和保持口腔卫生方面会有困难，并且咀嚼和吞咽食物也会更困难。我们正在探索促进中风患者口腔健康状况的不同方法。我们尤其关注您对治疗的感受以及治疗对您口腔、牙齿和日常生活的影响。

我们邀请您参加我们的研究。如果您同意参加，研究将包括以下治疗和评价：

首先，在您于医院接受中风治疗期间，我们将请您参加为期一周的临床研究。然后，在您出院一、三、六个月回医院进行常规复查时，我们将再对您做一次检查。

如果您同意参加，首先，口腔医生或研究助手会问您一些关于您健康状况、自我感觉以及您口腔和牙齿情况的问题。这些问题是世界各地同类研究中采用的常规问题。通过这些问题我们可以了解您的健康状况以及口腔问题对您的健康和生活品质的影响。我们将请您用一些带咸味的水漱口并吐在一个小杯里。然后我们会检查您的口腔、牙齿和牙龈。如果您有佩戴假牙的话，还将检查您的假牙。检查不会令您有不舒服的感觉，样本收集过程中也不会对您的身体造成任何不良的影响。您将会依照电脑随机分组被分配到以下两个治疗组中的一个。第一组，如何在日常生活中有效清洁牙齿和假牙(如果您有佩戴假牙的话)的专业建议；给予用电动牙刷和牙膏进行有效清洁牙齿和假牙（如果您有佩戴假牙的话）的专业建议，并在六个月内每天两次给予氯已定漱口液。当您在1，3，6个月复诊时，我们会再做一次相同的检查。

通过此项研究，我们希望能改善中风患者的口腔健康状况，并认识哪一种类型的治疗能最有效的降低脑卒中肺炎并发症的风险。我们将能够改善我们促进中风患者口腔健康的方法，协助中风患者康复并促进他们的全身健康状况。

副作用：使用氯已定漱口液没有严重的副作用，但可能会使牙齿暂时着色，这种着色可以刷牙去除。并且有的人可能会觉得氯已定漱口液有苦味。

任何时间您都可以中止治疗。

好处：以上两种治疗中的任意一种都能够直接地促进您的口腔健康，从而降低脑卒中肺炎并发症的风险。

隐私权：您提供的咨询将得到最严格的保密。研究结果可能会在科学会议或科学出版物上公布。但您的身份在任何形式的报告或出版物中都不会公开。

新的发现：在参与研究期间，您将会被告知有关研究的重要的新发现，这可能会影响您参加研究的意愿。

进一步的资讯请联络：

齐胤良，合肥市第二人民医院，安徽医科大学附属合肥医院，电话：189 5513 3681

非常感谢您的协助。

**参加研究专案同意书**

研究专案：**口腔健康促进对脑卒中肺炎并发症作用的研究**

研究实施者：合肥市第二人民医院，安徽医科大学附属合肥医院

以下条目用于检验您理解研究过程并同意参加此项研究：

1. 我确认我阅读并理解（或其他人为我阅读并说明）以上研究的资讯传单，并且留有备份。我有机会对研究专案的提问。我明白研究目的和存在的风险。
2. 我理解我的参加是自愿的。
3. 我同意参加此项研究。
4. 我明白我提供的所有资讯或我的医生向研究组传达的资讯是保密的，只有研究组能看到。
5. 我明白资料收集过程和给予资料的自愿性，我任何时候都可以收回研究组使用我的资料的权利而不给予理由，并且这将不会影响我的医疗和合法权利。
6. 我明白如果此项研究促成新的治疗方法或医学实验的发展，我将不会得到任何经济上的利益。
7. 我明白我任何时候都可以退出此项研究而不给予理由，并且这将不会影响我的医疗和合法权利。

请在以下同意书中签名：

患者或代理人： 日期： 签名：

检查者： 日期： 签名：

见证人： 日期： 签名：

如果患者同意参加而不能书写，代理人将代他/她签名
