# Supplementary material for: Using metagenomic analysis to assess the effectiveness of oral health promotion interventions in reducing risk for pneumonia among patients with stroke in acute phase: study protocol for a randomized controlled trial
Source: Trials. 2020 Jul 10;21:634. doi: 10.1186/s13063-020-04528-3 (PMC7350693; doi:10.1186/s13063-020-04528-3)
Supplement: Supplementary file 4 — Additional file 4. Translator’s certification and Anhui Province Natural Science Foundation Proposal of Funding Project. [file 13063_2020_4528_MOESM4_ESM.zip › Additional file 4_fuding 2R1.pdf]

Translator's Certification

I, Ruoxi Dai, have reviewed the Chinese documents in this application package; and have also reviewed the English translation of this document. I hereby attest that the English translation appears to be complete and correct. I am fluent in English and Chinese, and competent to translate the foreign language (Chinese) of this document into English.

Signature: Ruoxi Dai

Date: 4/11/2020

Cover:

Project Approval No.	1808085QH247

Anhui Province Natural Science Foundation

Proposal of Funding Project

Funding category: Youth Funding Project

Project title: Research on the effectiveness of oral health promotion interventions on post stroke complication

Project duration: 07/2018 – 06/2020

Principal Investigator: DAI, RUOXI

Supporting institution: Anhui Medical University

Version made by Anhui Province Natural Science Foundation committee office

2010

项目编号：	1808085QH247

安徽省自然科学基金项目

计 划 任 务 书

资 助 类 别： 青年项目

项 目 名 称： 口腔健康促进对脑卒中肺炎并发症作用的研究

起 止 时 间： 201807 - 202006

主 持 人： 戴若曦

归口管理单位： 安徽医科大学

安徽省自然科学基金委员会办公室制

二〇一〇年

填写说明

1、本任务书由省自然科学基金委员会办公室（以下简称省基金办）指导填写。

2、填写《计划书》时要求科学严谨、实事求是、表述清晰、准确。《计划书》经项目主管部门审核批准后，将作为项目拨款、研究计划执行、检查和验收的依据。

3、资助类别指面上基金或青年基金。

4、主持人指项目第一申请人。

5、课题组成员指除申请者（即项目主持人）之外的其它成员。

6、项目组成员和研究内容按申请书执行，一般不得修改。

本计划任务书所称<u>**口腔健康促进对脑卒中肺炎并发症作用的研究**</u>项目，由科基 [2018] 17 号文批准立项。为规范项目实施和管理，项目主管机关省基金办和项目主持人及项目管理单位签订本计划任务书，共同遵照执行。

本项目基本表述和规定以申报书和计划文件为准，本任务书条款另有规定的按本任务书执行。

一、项目基本信息

项目信息	项目名称	口腔健康促进对脑卒中肺炎并发症作用的研究						
	资助类别	青年项目			执行年限	2		
	学科领域	口腔颌面疾病其他科学问题			资助金额	10.0		
主持人信息	姓名	戴若曦	性别	女	出生年月	1985-12	民族	汉族
	学位	博士			职称	讲师		
	电话	0551-65167732			手机			
	电子邮件	dairuoxi@gmail.com						
	工作单位	安徽医科大学						
	通讯地址	安徽省合肥市梅山路 81 号						
依托单位信息	名称	安徽医科大学						
	联系人	吕红霞		电子邮件	hxjj2010@126.com			
	电话	0551-65167731		手　机				

课题组成员（不含主持人）	姓　名	性别	出生年月	技术职称	所 在 单 位	签 名
	卢展民	男	1960-03	高级	香港大学	卢展民
	戴元昶	男	1957-10	高级	安徽医科大学	戴元昶
	蒋勇	男	1962-10	高级	安徽医科大学	蒋勇
	齐胤良	男	1986-01	初级	安徽医科大学	齐胤良
	瞿明胜	男	1985-06	中级	安徽医科大学	瞿明胜

	王晴晴	女	1991-05	其它	安徽医科大学	王晴晴

AHNSF

二、主要研究内容、预期目标及拟解决的关键问题

主要研究内容：

本项目的总体思路：通过循证医学的"金标准"— 随机对照试验研究，对纳入的脑卒中患者进行多次跟踪随访，评估本研究设计的口腔健康促进干预措施，即高级口腔卫生保健对脑卒中综合治疗的作用。一方面，在临床层面上，评价高级口腔卫生保健是否显著降低口腔菌斑水平和改善口腔健康状况，降低脑卒中肺炎并发症，提高脑卒中患者的生命质量；另一方面，通过实验室研究，在微生物群落水平上，明确脑卒中肺炎并发症患者口腔样本中的微生物与呼吸道吸痰样本中微生物在种类上是否具有一致性。明确高级口腔卫生保健是如何通过控制"口腔机会致病微生物"和"肺部致病微生物"的种类、携带率以及携带量，达到降低脑卒中肺炎并发症的发生。

1. 口腔健康促进对脑卒中病人作用的随机对照试验研究

本部分使用平行、对照、随机的试验设计，对研究对象进行多次跟踪随访,评估本项目设计的高级口腔卫生保健干预措施：

①对脑卒中病人口腔菌斑水平和其它口腔健康状况的作用；

②对脑卒中病人肺炎并发症发生的作用；

③对脑卒中病人生命质量的作用。

2. 口腔健康促进对与脑卒中肺炎并发症相关的口腔机会致病微生物作用的实验室研究

本部分微生物学研究：

① 通过获取所有研究对象中脑卒中肺炎并发症患者的口腔漱口样本和呼吸道吸痰样本，比较肺炎并发症发生前"口腔机会致病微生物"和肺炎并发症发生后的"肺部致病微生物"在种类上的多样性和相似性；

② 比较干预组和对照组的口腔漱口样本，展示干预措施对"口腔机会致病微生物"的种类、携带率、携带量的作用；

③ 比较干预组和对照组中肺炎并发症患者的呼吸道吸痰样本，展示干预措施对"肺部致病微生物"的种类、携带率、携带量的作用；

④ 比较干预组内发生肺炎患者和无肺炎发生患者的漱口样本，展示干预措施在控

制“口腔机会致病微生物”上的异质性；

⑤ 比较干预组内肺炎并发症患者和无肺炎发生患者的呼吸道吸痰样本，展示干预措施对“肺部致病微生物”上的异质性。

3. 综合判断口腔健康促进是否具有常规纳入脑卒中综合治疗的价值

通过上述研究，评估口腔健康促进对研究对象的临床健康的客观指标和生命质量的主观指标，及其在微生物病原学层面上的作用，建立三者之间的关联性，完善评价口腔健康促进有效性的精确客观体系。最终综合判断口腔卫生保健常规纳入脑卒中综合治疗的价值。

预期目标：

综合判断口腔健康促进是否具有常规纳入脑卒中综合治疗的价值：

1. 临床水平上，明确口腔健康促进是否可以降低脑卒中肺炎并发症的发生和提高脑卒中病人的生命质量；

2. 微生物水平上，甄选出参与肺炎发生的口腔机会致病微生物的种类，明确口腔健康促进对其携带率和携带量的作用。

拟解决的关键问题：

1. 脑卒中肺炎并发症和口腔机会致病微生物的相关性

获取肺炎发生前口腔漱口样本和肺炎发生后呼吸道吸痰样本，运用微生物学研究比较“口腔机会致病微生物”和“肺部致病微生物”在种类上的多样性和相似性，甄选出参与肺炎发生的“口腔机会致病微生物组”。

2. 口腔健康促进干预措施对“口腔机会致病微生物组”的携带率和携带量的作用

通过比较干预组和对照组的口腔漱口样本，以及比较干预组和对照组中肺炎并发症患者的呼吸道吸痰样本，从而揭示干预措施对“口腔机会致病微生物组”的携带率和携带量的作用。

三、年度计划内容与阶段目标（须根据项目总体考核指标内容填写阶段考核指标）

	年度实施内容和考核指标
2018 年 7 月至<br>2018 年 12 月	1. 完成基线招募并获得知情同意；<br>2. 完成基线临床口腔检查，基线问卷评估，基线采集，实施干预措施；<br>3. 根据基线数据对脑卒中患者进行关于脑卒中肺炎症、临床口腔健康、生命质量的横断面分析
2019 年 1 月至<br>2019 年 12 月	1. 完成对研究对象的随访评估：临床口腔检查，问卷评估，肺炎并发症发生的跟踪，样本采集；<br>2. 样本培养和菌种保存；<br>3. 根据随访评估数据，分析干预组是否显著性地提高临床口腔健康、降低肺炎发生、提高生命质量<br>4. 参加国内/国际会议，发表国内期刊文章 2 篇
2020 年 1 月<br>2020 年 6 月	1. 菌种鉴定；<br>2. 统计模型建立和分析<br>3. 发表 SCI 论文 2 篇

四、预期研究成果和主要考核指标

（包括：发表高层次学术论文（论著）、争取国家级项目资助、取得自主知识产权及培育应用性成果等）

1. 甄选出可能参与肺炎发生的“口腔机会致病微生物”的种类；
2. 揭示口腔干预措施是否可以控制“口腔机会致病微生”和“肺部致病微生”的种类、携带率、携带量；
3. 参加国内/国际会议，发表国内核心期刊文章 2 篇，SCI 论文 2 篇

五、经费计划

单位：万元

经费预算	总经费：10.0		
	自　筹　经　费	省财政资助经费	其它渠道经费
	0.0	10.0	0.0

年度 / 来源	2018　年	2019　年	2020　年
省财政拨款	10.00	0.00	
自　　筹	0	0	0

省财政拨款开支预算

科　　目	金　额	备　　注
（一）直接费用	7.7	一、直接费用<br>1. 材料费<br>（1）口腔卫生保健用品费用<br>共计 1.5 万<br>电动牙刷：对照组脑卒中患者将在 6 个月内被提供 1 支电动牙刷和一支更换刷头，万：50 元×100 人；0.2%葡萄糖酸漱口水：对照组组中脑卒中患者被提供 6 个月用量的 0.2%氯己定漱口水，万：100 元×100 人<br>（2）口腔临床检查器械和耗材费<br>共计 0.4 万<br>器械：WHO 牙周探针，0.1 万：100 元×10 支；可携带灯光口镜 0.2 万：1000 元×2 支；耗材，0.1 万：一次性手套、口罩、检查衣<br>（3）实验室耗材<br>共计 0.1 万<br>存储管、移液器、吸头等<br>（4）实验室培养基、生化试剂盒费用<br>共计 2.0 万
1. 设备费	0	
2. 材料费	4	
3. 测试化验加工费	1	
4. 燃料动力费	0	
5. 差旅费	0.8	
6. 会议费	0.6	
7、国际合作与交流费	0	
8、出版/文献/信息传播/知识产权事务费	0.8	
9、劳务费	0.5	
10、专家咨询费	0	
11、其他支出	0	
（二）间接费用	2.3	

其中：绩效支出	0. 38	2. 测试加工费用<br>用于不可通过生化试剂盒鉴定出的细菌样本，需要进行 DNA 测序鉴定，共计 1.0 万<br><br>3. 差旅费<br>国际会议差旅费 0.8 万<br><br>4. 会议费<br>国际会议报名费用 0.6 万<br><br>5.出版/文献/信息/传播/知识产权事务费<br>SCI 文章版面刊出费用 0.8 万<br><br>5. 劳务费<br>主要用于支付参与项目研究生的津贴，一名研究生 250 元/人×20 个月×1 人=0.5 万元

五、管理条款

省基金办和项目主持人及项目管理单位按照《安徽省自然科学基金管理办法》管理、监督和组织实施。

（一）主持人（申请人）及课题组：

1、遵守安徽省自然科学基金的有关管理规定，按照计划文件及任务书确定的研究内容和工作进度抓紧项目的实施工作；

2、遇有问题及时向本单位的科研业务管理部门报告，争取尽快解决；

3、每年年底向省基金办提供一份项目研究进展报告；

4、阶段成果或最终成果发表的论文或参加学术会议等的文字材料应注明"安徽省自然科学基金资助项目，项目编号：×××××"，同时抄送省基金办一份；

5、按计划完成工作，及时申请项目结题（验收或评价）。

（二）项目管理单位：

1、给予项目课题组必要的条件及匹配资金等支持；

2、协助省基金办组织、督促与协调项目实施；

3、监督资助经费的使用；

4、对项目执行中重大事项（如目标、人员调整或无法正常执行的项目），向省基金办书面提出处理建议；

（三）省基金办：

1、按照计划及项目进展情况及时拨付资助经费；

2、检查计划项目的实施情况，对重点项目组织阶段评估；

3、根据项目执行情况决定项目的调整、结转、暂缓执行或终止执行等；

4、组织项目结题（验收或评价）。

本计划任务书一式四份，主持人和其所在单位或归口管理单位各一份、省基金办两份。

AHNSF

省基金办

经办人（签字）：

负责人（签字）：

（公章）：　　　　　　　　年　　月　　日

主持人

（签字）：　　　　　　　　2018 年 7 月 11 日

项目所在单位（归口管理部门）

负责人（签字）：

（公章）

开户银行：

帐　　号：　　　　　　　　2018 年 7 月 13 日
