# Supplementary material for: Using metagenomic analysis to assess the effectiveness of oral health promotion interventions in reducing risk for pneumonia among patients with stroke in acute phase: study protocol for a randomized controlled trial
Source: Trials. 2020 Jul 10;21:634. doi: 10.1186/s13063-020-04528-3 (PMC7350693; doi:10.1186/s13063-020-04528-3)
Supplement: Supplementary file 4 — Additional file 4. Translator’s certification and Anhui Province Natural Science Foundation Proposal of Funding Project. [file 13063_2020_4528_MOESM4_ESM.zip › Addtional file 4_fundingR1.pdf]

CALIFORNIA JURAT WITH AFFIANT STATEMENT **GOVERNMENT CODE § 8202**

[X] See Attached Document (Notary to cross out lines 1–6 below)
[ ] See Statement Below (Lines 1–6 to be completed only by document signer[s], *not* Notary)

Signature of Document Signer No. 1 *Signature of Document Signer No. 2 (if any)*

A notary public or other officer completing this certificate verifies only the identity of the individual who signed the document to which this certificate is attached, and not the truthfulness, accuracy, or validity of that document.

State of California
County of San Diego

Subscribed and sworn to (or affirmed) before me on this 7th day of December, 2017, by
Date *Month* *Year*

(1) Yu Zhao

(and (2) ____________),
Name(s) of Signer(s)

proved to me on the basis of satisfactory evidence to be the person(s) who appeared before me.

OFFICIAL SEAL
KRISTALEE ATKINS
NOTARY PUBLIC-CALIFORNIA
COMM. NO. 2120228
SAN DIEGO COUNTY
MY COMM. EXP. JULY 20, 2019

Signature [signature]
Signature of Notary Public

Seal
Place Notary Seal Above

OPTIONAL

Though this section is optional, completing this information can deter alteration of the document or fraudulent reattachment of this form to an unintended document.

Description of Attached Document

Title or Type of Document: Translators Declaration **Document Date:** 12/7/2017

Number of Pages: 3 **Signer(s) Other Than Named Above:** ____________

©2014 National Notary Association • www.NationalNotary.org • 1-800-US NOTARY (1-800-876-6827) Item #5910

ata MEMBER
American Translators Association

TRANSLATOR'S DECLARATION

STATE OF CALIFORNIA

COUNTY OF SAN DIEGO

I, Yu Zhao, declare that I am a member of the American Translators Association (member number 265081), am fluent in English and Chinese, can translate from Chinese to English and from English to Chinese. I further declare that I have translated the document stated below and which is attached to this declaration:

Proposal of Funding Project
(Title of Document)

I declare that, to the best of my knowledge and abilities, the attached document in English is a true and accurate translation of the attached document in Chinese .

I declare under penalty of perjury under the laws of the State of California that the foregoing is true and correct.

This declaration is signed this 7th day of December, 2017, in Oceanside, California, U.S.A.

____
(Signature of Translator)

Yu Zhao
____
(Print Name)

Project Approval No.	81701036
Project Code:	H1411
Centralized Management Unit:	
Supporting Institution Code:	23003208A0017-0029

817010361010830.00

National Natural Science Foundation of China

Proposal of Funding Project

Funding category: Youth Science Funding Project

Subclass description:

Notes:

Project title: Research of the Effect of Oral Health Promotion on Pneumonia Complication Stroke

Direct cost: 190,000 RMB　Project duration: Jan, 2018 - Dec, 2020

Principal: DAI, RUOXI

Mailing address: 81 Meixi Road, Hefei City, Anhui Province

Zip code: 230032　Telephone: 0551-65118677

Email: dairuoxi@gmail.com

Supporting institution Anhui Medical University

Contact person: Ke, Daoping　Telephone: 0551-65161053

Application date: August 22, 2017

Under the Supervision of National Natural Science Foundation of China

Version: L010830

项目批准号	81701036
申请代码	H1411
归口管理部门	
依托单位代码	23003208A0017-0029

81701036101 0830

国家自然科学基金委员会

资助项目计划书

资助类别：青年科学基金项目

亚类说明：

附注说明：

项目名称：口腔健康促进对脑卒中肺炎并发症作用的研究

直接费用：19万元　　执行年限：2018.01-2020.12

负 责 人：戴若曦

通讯地址：安徽省合肥市梅山路81号

邮政编码：230032　　电　　话：0551-65118677

电子邮件：dairuoxi@gmail.com

依托单位：安徽医科大学

联 系 人：柯道平　　电　　话：0551-65161053

填表日期：2017年08月22日

国家自然科学基金委员会制

Version: 1.010.830
